# Estimating the probability of demonstrating vaccine efficacy in the declining Ebola epidemic: a Bayesian modelling approach

**DOI:** 10.1136/bmjopen-2015-009346

**Published:** 2015-12-15

**Authors:** Anton Camacho, Rosalind M Eggo, Sebastian Funk, Conall H Watson, Adam J Kucharski, W John Edmunds

**Affiliations:** Department of Infectious Disease Epidemiology, London School of Hygiene & Tropical Medicine, London, UK

**Keywords:** Clinical trials < THERAPEUTICS, STATISTICS & RESEARCH METHODS, Protocols & guidelines < HEALTH SERVICES ADMINISTRATION & MANAGEMENT

## Abstract

**Objectives:**

We investigate the chance of demonstrating Ebola vaccine efficacy in an individually randomised controlled trial implemented in the declining epidemic of Forécariah prefecture, Guinea.

**Methods:**

We extend a previously published dynamic transmission model to include a simulated individually randomised controlled trial of 100 000 participants. Using Bayesian methods, we fit the model to Ebola case incidence before a trial and forecast the expected dynamics until disease elimination. We simulate trials under these forecasts and test potential start dates and rollout schemes to assess power to detect efficacy, and bias in vaccine efficacy estimates that may be introduced.

**Results:**

Under realistic assumptions, we found that a trial of 100 000 participants starting after 1 August had less than 5% chance of having enough cases to detect vaccine efficacy. In particular, gradual recruitment precludes detection of vaccine efficacy because the epidemic is likely to go extinct before enough participants are recruited. Exclusion of early cases in either arm of the trial creates bias in vaccine efficacy estimates.

**Conclusions:**

The very low Ebola virus disease incidence in Forécariah prefecture means any individually randomised controlled trial implemented there is unlikely to be successful, unless there is a substantial increase in the number of cases.

**Strengths and limitations of this study**

- Timely estimates of chance of success of individually randomised controlled trials (RCTs) in the declining Ebola epidemic.
- Determination and explanation of bias introduced to vaccine RCTs by exclusion of cases that occur shortly after vaccination.
- This model can only account for RCTs conducted in the declining phase of the epidemic.

## Introduction

Since 2013, the largest epidemic of Ebola virus disease (EVD) to date has been ongoing in West Africa, with over 25 000 cases and 10 000 deaths reported as of 7 July 2015. There is no licensed vaccine or treatment for EVD, and the case fatality rate is around 70%.^1^ The epidemic has declined since its peak, however disease incidence remains low.^2^ As a result, it may be challenging to run the phase III vaccine trials necessary to assess the efficacy of candidate vaccines that are currently in development, and hence apply for licensure. As well as existing study designs being proposed, such as individually randomised controlled trials (RCTs) and stepped wedge trials,^3^ the declining incidence of EVD has led to development of novel vaccine trial designs^4^ to account for the limited number of cases in West Africa.

Some areas have continued transmission, however, and thus remain potential candidate locations for a large-scale Ebola vaccine trial.^5^ For example, trials have been proposed in Guinea, where Forécariah prefecture has seen continuing transmission since October 2014. Conventional statistical power analysis or sample size estimation using fixed assumptions on incidence rates is inappropriate when incidence rates change during the course of an epidemic. Here we use a combination of epidemic modelling and statistical analysis to examine the chance of success of such trials. Specifically, we estimate the power of an RCT to detect vaccine efficacy in the coming months under a range of different scenarios. Unlike other approaches,^6^ our method uses real-time forecasting to account for the possibility that the epidemic will end during the trial, and incorporates this possibility into the evaluation of trial success.

## Methods

### Model fitting and forecasting

To investigate the dynamics of EVD in the prefecture of Forécariah (population 245 000), we fitted a stochastic Susceptible-Exposed-Infectious-Recovered (SEIR) transmission model to the weekly incidence of confirmed and probable cases published by the WHO^7^ and Guinean Ministry of Health between 1 August 2014 and 7 June 2015.^2^ A description of parameters is given in table 1.

**Table 1:** Parameter descriptions and values

Parameter	Description	Value
β_t_	Time varying transmission rate	Estimated
1/ɛ	Average latent period	9.4 days^1^
1/ν	Average infectious period	11.8 days^1^
*R*_t_	Time-varying reproduction number	β_t_/ν
1/κ	Average time between vaccination and protection	14 days
1/γ	Average duration of vaccine protection	1 year
σ	Vaccine efficacy	0%, 50%, 70%, 90%

We used published estimates of 9.4 days for the mean latent period and 11.8 days for the mean infectious period.^1^ To account for external influences on the reproduction number (*R*_t_), for example, variation in population behaviour, or epidemic control measures, we assumed that the transmission rate could change over time. Therefore, the change in β_t_ would also absorb any effective change in the infectious period during the epidemic. The extent and direction of rate change was estimated during the model-fitting procedure.^8^ We used the same Bayesian inference framework as in.^9^ Briefly, we defined the likelihood of the data through a negative binomial observation process accounting for over-dispersion in the reporting of cases (the mean reporting rate was fixed at 60% and the dispersion parameter was inferred). Then, we used a particle Monte-Carlo Markov Chain^10^ algorithm implemented in the SSM library^11^ to sample from the marginal posterior distribution of the parameters and the states of the model.

**Figure 1:**
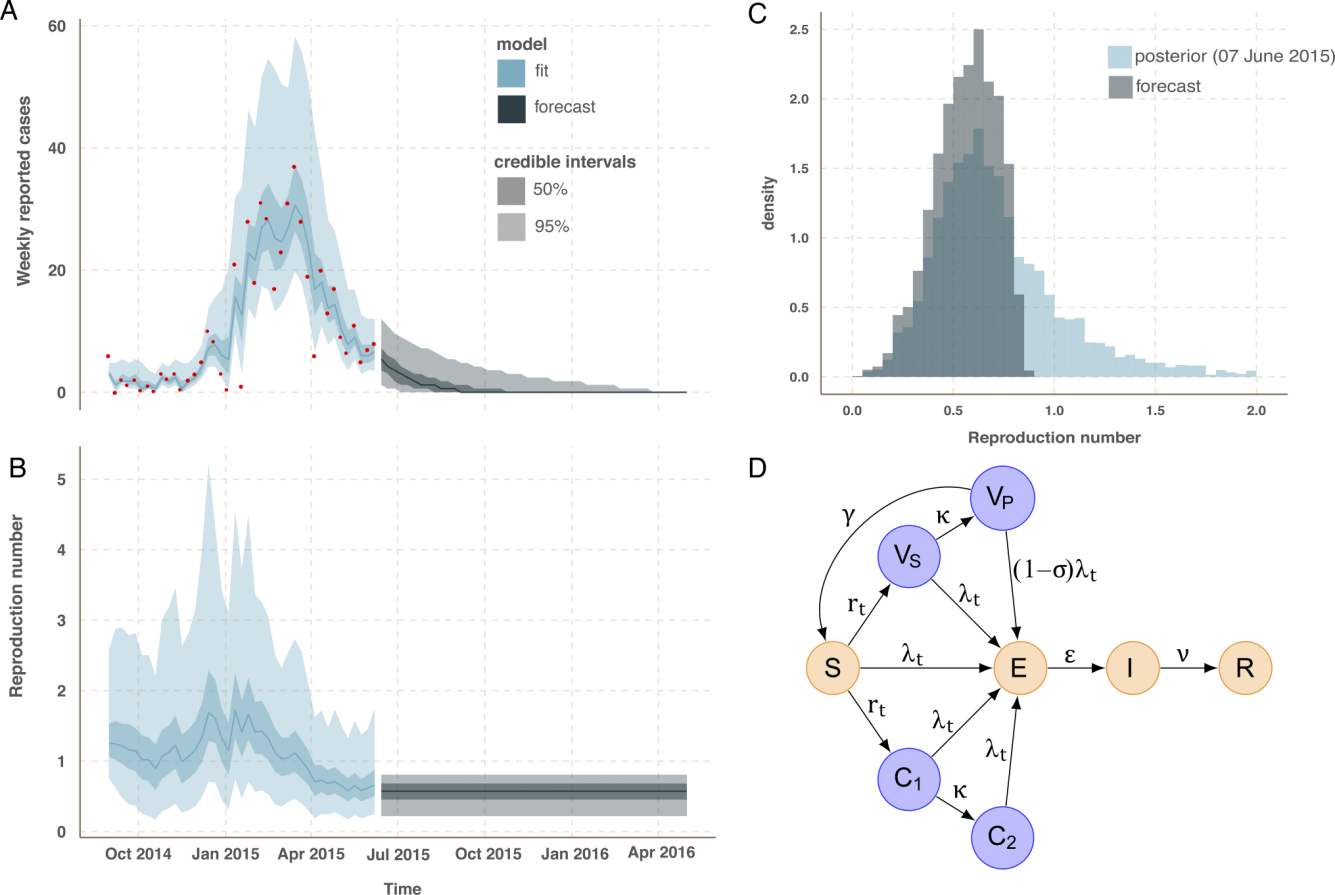
Model fit (blue) to the incidence data in Forécariah (red points) and forecast (grey) based on the posterior distribution at the latest data point (A). The solid line corresponds to the median estimate and the shaded areas to the 50% and 95% credible intervals. Fitted (blue) and forecasted (grey) values for the time-varying reproduction number *R*_t_ (B). Posterior distribution of *R*_t_ on 7 June 2015 (blue) and distribution corresponding to the trajectories used for forecasting (grey)(C). Mechanistic model for the vaccine trial (D). Susceptible participants are recruited into the trial at rate, rt. Before the trial begins, rt equals zero, and the model reduces to an SEIR model. Those entering the vaccine arm pass through a period of immune development, V_s_, during which they are susceptible to Ebola virus disease infection. Following onset of protective immunity, they enter V_p_ and experience reduced susceptibility, σ, equal to the vaccine efficacy. Protective immunity is lost at rate γ, and individuals become susceptible again. Participants enter the control arm at the same rate as vaccinated participants and are separated between early (C_1_) and late (C_2_) control to match the delay in acquiring immunity in the vaccine arm. For biological realism, the distribution of durations of E, V_s_ and V_p_ follow an Erlang distribution with shape parameter two. Similarly, to match the vaccine arm, the same distribution is assumed for the compartment C_1_.

After fitting the transmission parameters of the model, we projected the model forward in time, to simulate the potential future trajectories of the epidemic. More precisely, we simulated 200 000 epidemic trajectories without a vaccine trial from 7 June 2015 until 1 May 2016. This corresponds to 40 stochastic simulations of 5000 samples from the posterior distribution of the parameters and model states inferred on 7 June 2015. We restricted the forecast to those parameter sets for which more than 25% of the 40 simulated epidemics go extinct before 1 May 2016, that is, assuming that elimination of EVD will be achieved within 10 months. We kept 3542 (71%) of the 5000 parameter sets. Epidemic trajectories resulting from these parameter sets are summarised in figure 1A, and the distribution of *R*_t_ for forecasted epidemics is shown in figure 1B, C. In particular, we note that all forecasts have *R*_t_ below the epidemic control threshold, that is, we assume that the epidemic will remain under control until elimination. This is a reasonable assumption, given the low incidence in Forécariah.

### Trial implementation

To model the vaccine trial, we extend the stochastic SEIR transmission model to include the recruitment of two arms of an individually randomised controlled trial^12^ for an EVD vaccine (figure 1D).

In the model, the vaccine is delivered in one dose, and protective immunity begins 2 weeks later. We also conducted a sensitivity analysis by using 1 week delay, based on the intermediate results of the rVSV ring-vaccination trial in Guinea.^13^ Immunity lasts 1 year and has efficacy of assumed values 0%, 50%, 70% or 90%. Each arm of the modelled trial has 50 000 participants, and we test three potential start dates: 1 July, 1 August and 1 September 2015, and we also test two modes of recruitment; immediate, where 100 000 individuals are recruited in the first 2 weeks of the trial and gradual, where 10 000 individuals are recruited during the first 2 weeks of each month, for 10 months. The gradual recruitment scenario is more realistic because of the delays inherent in recruiting and vaccinating people, however we present the immediate recruitment scenario as an example of the ideal implementation.

### Analysis of trial outcomes

In a primary analysis of a randomised controlled vaccine trial, the vaccine efficacy, 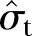, at time t is measured by:
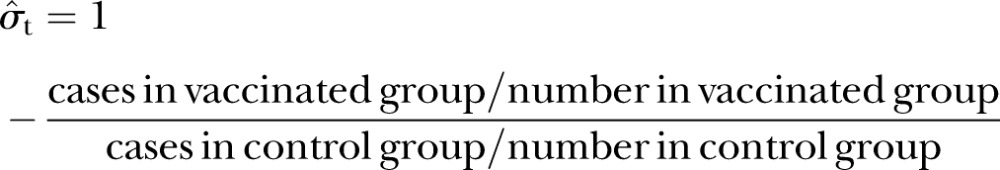
where the vaccinated and control groups are defined in the trial protocol (see below), and 95% CIs are computed as score CIs.^14^ These standard vaccine efficacy calculations assume that the risk of infection is constant through time in both arms, which is violated when an epidemic is declining. Forécariah has seen unstable declining incidence since mid-March 2015 (figure 1A), which has two implications for a trial in this area; (1) the false-positive rate (type I error) may be different than the expected 5%, and, (2) the trial may be under-powered if the epidemic goes extinct before enough events have occurred (type II error).

For each trial simulation, we computed 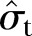 each week until the simulated epidemic went extinct. A positive (negative) effect is defined if the lower (upper) bound of the CI is strictly positive (negative). We then derived:

- Extinction probability—the probability that the epidemic has gone extinct by time t, which is the proportion of extinct simulations at time t.
- Measured vaccine efficacy—the median value of 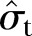 in simulations where the epidemic is non-extinct at time t and at least one case occurred in either the control or intervention arms (otherwise 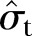 is not defined).
- False-positive rate—the probability that a positive or negative vaccine effect can be detected, given that the vaccine has no efficacy and the epidemic is non-extinct at that time. Calculated as the proportion of simulations with a positive or negative vaccine efficacy when true efficacy is 0%, and the epidemic is non-extinct at t.
- Power to detect vaccine efficacy—the probability that a positive vaccine effect can be detected given that the vaccine is efficacious and the epidemic is non-extinct. We use the proportion of simulations with a positive effect among the non-extinct simulations at time t.
- Power adjusted by extinction probability—the probability that the epidemic is non-extinct and vaccine efficacy is detected. The power at time t is multiplied by 1-extinction probability at time t, and this therefore represents the chance of success of the trial.

### Definition of the vaccine and control groups

Some trial protocols exclude participants who develop symptoms shortly after vaccination, that is, before the vaccine becomes immunoprotective, under the assumption that the participant became infected before recruitment or before the vaccine could generate an immune response in the host. Other trial protocols also exclude control participants who develop symptoms within this period,^12^ under similar assumptions about exposure time. We test the effect of excluding participants in each arm who are infected within 2 weeks of recruitment. We distinguish three definitions of the vaccinated or control groups depending on which participants are excluded: (1) no early cases excluded, (2) only early cases in the vaccine arm are excluded, (3) early cases in both arms are excluded.

## Results

### Detection of vaccine efficacy

**Figure 2:**
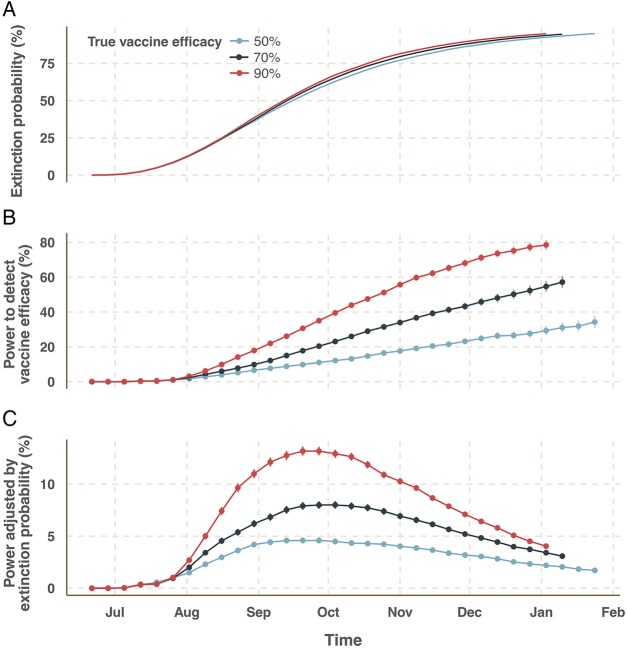
Detection of vaccine efficacy for a trial starting on 1 July with immediate vaccination. Extinction probability (A), power to detect efficacy (B) and power to detect efficacy adjusted by extinction probability (C), for assumed efficacy values 50, 70 and 90%.

For each vaccine efficacy tested, the extinction probability quickly increases through time, with more than 50% chance of extinction by October 2015 (figure 2A). Power is positively correlated with vaccine efficacy (figure 2B). The power to detect vaccine efficacy at a given time depends on the probability that the epidemic has not gone extinct by that time, which is also influenced by the true vaccine efficacy (*σ*), due to population-level immunity caused by the vaccine trial. Figure 2C shows that there is low power to detect efficacy when adjusted by the extinction probability.

### Effect of vaccine or control group definition

**Figure 3:**
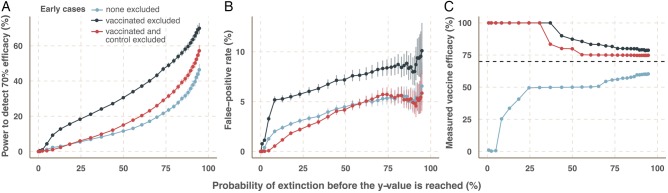
Effect of group definition on trial outcomes. The X-axis shows the probability that the epidemic goes extinct before the value on the Y-axis is reached. Advancing time moves left-to-right, as the extinction probability increases. Power to detect efficacy (A), false-positive rate (B), and measured vaccine efficacy (where 70% is assumed) (C). The three group definitions (1) no early cases excluded (blue), (2) early cases in the vaccine arm excluded (black), and (3) cases in both arms excluded (red).

For a model with immediate recruitment on 1 July, and 70% vaccine efficacy, the highest power is achieved by excluding only early cases in the vaccine arm (figure 3A). However, this cohort definition is also associated with an inflated type I error (figure 3B) and overestimates the true vaccine efficacy (figure 3C). Excluded cases have a higher risk of infection because the force of infection decreases over time. Since early cases in the control arm that were at the same high risk are not excluded, bias is introduced.

Including all cases reduces the false-positive rate below 5% but also decreases the trial power, and leads to underestimates of vaccine efficacy (figure 3C). Excluding early cases in vaccinated and control arms maintains the false-positive rate below 5%, does not bias vaccine efficacy downward and maintains moderate power. In all cases, there is high probability that the epidemic goes extinct before vaccine efficacy can be accurately measured. These results are generalisable to other vaccine efficacies and start dates.

**Figure 4:**
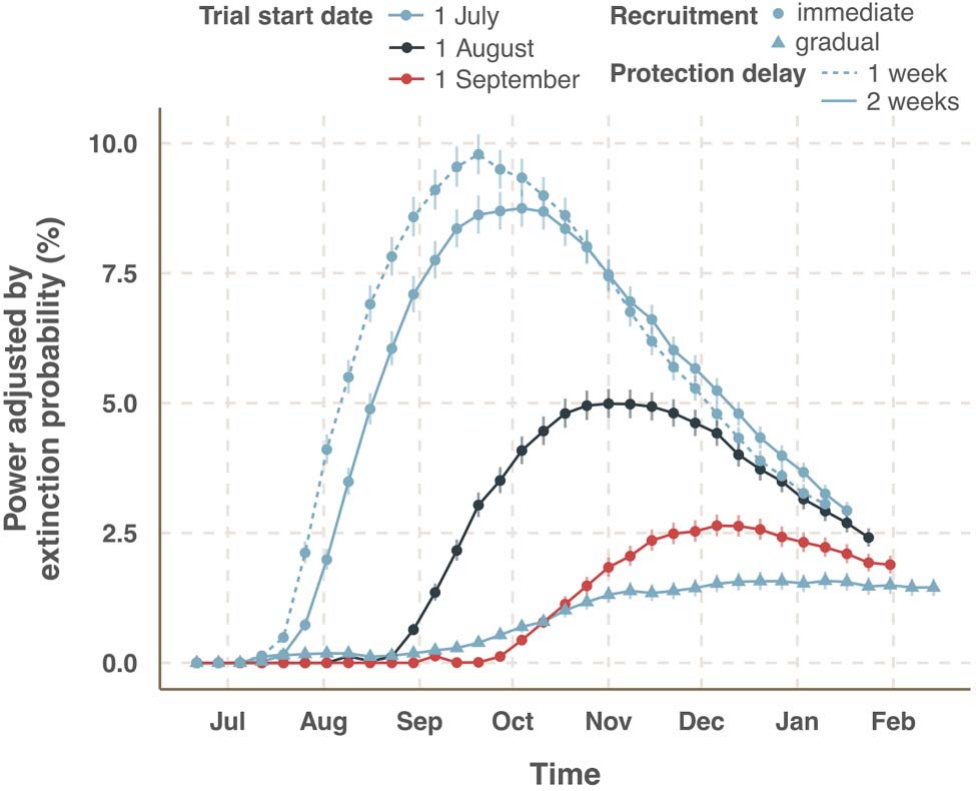
Effect of start date on trial success. The figure shows immediate administration of a 70% efficacious vaccine to all participants (round points) for trials starting on the 1 July, 1 August and 1 September. In addition, the gradual recruitment of participants (triangles) is shown for the 1 July start date. The dashed line shows the power when the assumed delay from administration of vaccine until protective immunity is 1 week. All other results are for a 2-week delay.

In practice, it may be difficult to find the appropriate exclusion period, where the period over which immunity is developed is unknown. Reducing both the protection delay and exclusion period from 2 to 1 week leads only to a slightly earlier and higher peak in power due to greater sample sizes and number of cases included in the analysis. In addition, shorter delay increases herd immunity effect, leads to faster extinction of the epidemic and thus reduces the adjusted power at later time (figure 4).

### Realistic rollout scenarios

Under an ideal scenario of immediate recruitment of 100 000 participants, the later the trial starts the lower the probability to detect vaccine efficacy (figure 4). The effect of delaying the start of the trial by 1 month halves the chance of success. Gradual enrolment of participants drastically reduces the power to detect efficacy compared with immediate rollout. The example shown is 1 July start date although other start dates show the same pattern, with greatly reduced power. This occurs because there is a high probability of extinction before enough participants are recruited.

## Discussion

Here we modelled the implementation of an individually randomised control vaccine trial in Forécariah prefecture, Guinea using an extended version of a previously published dynamic transmission model for EVD. We showed that if an RCT were to start later this year in Forécariah prefecture it would have a very limited chance of detecting any vaccine efficacy, because the epidemic is likely to go extinct before enough cases have occurred in participants. In addition, in realistic rollout scenarios of 10 000 participants per month, the chance that the epidemic persists until enough participants are recruited and the trial is able to detect efficacy is very low, for example, below 2% for a trial beginning on 1 July 2015 with a 70% efficacious vaccine. We note that this adjusted power is probably an overestimate since our model operates at the population level and does not account for clustering effect at small scales.

We also demonstrated that exclusion of early cases in the group definition for the vaccine arm of a trial (ie, individuals vaccinated but not yet protected) inflates the power but also the false-positive rate due to the declining risk of infection over time. Ideally, the group definition for a primary analysis in a declining epidemic should consider excluding early cases in the control and intervention arms to maximise the power to detect vaccine efficacy while keeping the false-positive rate below 5%. Alternatively, more advanced statistical analyses accounting for time-varying risk of infection should be considered to circumvent the necessity of excluding early control cases.^6^ This important bias must be accounted for in protocols of infectious disease vaccine trials.

Overall, our analysis is an example of how real-time mathematical models can be used to design trials more efficiently during an epidemic, and assess feasibility of planned trials, although models are infrequently utilised to this end. More realistic models accounting for network structure could be even more precise given that the majority of transmission events may be seen in clusters formed at confined space (eg, hospital or household) and also at a small spatial scale.
